# Some citation-related characteristics of scientific journals published in individual countries

**DOI:** 10.1007/s11192-013-1053-1

**Published:** 2013-05-31

**Authors:** Keshra Sangwal

**Affiliations:** Department of Applied Physics, Lublin University of Technology, ul. Nadbystrzycka 38, 20-618 Lublin, Poland

**Keywords:** Impact factors, Journal self-citations, Journal categories, Journal language

## Abstract

Relationships between publication language, impact factors and self-citations of journals published in individual countries, eight from Europe and one from South America (Brazil), are analyzed using bibliometric data from Thomson Reuters JCR Science Edition databases of ISI Web of Knowledge. It was found that: (1) English-language journals, as a rule, have higher impact factors than non-English-language journals, (2) all countries investigated in this study have journals with very high self-citations but the proportion of journals with high self-citations with reference to the total number of journals published in different countries varies enormously, (3) there are relatively high percentages of low self-citations in high subject-category journals published in English as well as non-English journals but national-language journals have higher self-citations than English-language journals, and (4) irrespective of the publication language, journals devoted to very specialized scientific disciplines, such as electrical and electronic engineering, metallurgy, environmental engineering, surgery, general and internal medicine, pharmacology and pharmacy, gynecology, entomology and multidisciplinary engineering, have high self-citations.

## Introduction

Different aspects of the scientific publication behaviour of researchers publishing in various national and international journals have been studied and differences between the citations of papers in English and non-English languages on a global level have been recognized. Several studies have shown that citations per paper of non-English journals are lower than those of English journals (Garfield 1978; Gonzalez-Alcaide et al. 2012; Liang et al. 2013; Mueller et al. 2006; Poomkottayil et al. 2011; Sangwal 2012; van Raan et al. 2011). Campbell (1990) found that US and UK researchers have a tendency to cite publications produced in their own countries. There are also evidences that researchers are more likely to cite papers published in national languages when publishing in national journals than in international journals (Garfield 1978; Liang et al. 2013; Lin and Zhang 2007). Language self-citation has been suggested as the primary cause of this biased citation behavior in these journals (Liang et al. 2013). Sangwal (2012) analyzed the publication trends of Polish professors and found that: the citability of papers published by physics, chemistry and technical sciences professors in Poland decreases with increasing fraction of the papers in volumes/issues of journals as proceedings of conferences and in non-English language journals.

The scientific impact of journals is traditionally measured in terms of their impact factors (IFs) calculated from the total number of citations, including self-citations, received by the papers published in them and the ranking of a journal in its scientific discipline is determined by the journal IF. These IFs of journals are usually used by research funding agencies as an evaluation measure of scientific performance of individual researchers, faculties and institutes. For example, the Polish Ministry of Higher Education has introduced a system of funding of research in university faculties and institutes and independent research institutes based on their categories determined from consideration of their scientific research outputs. The categories of the research units are determined according to a standardized evaluation criterion based on the number of points assigned to different publications of their publication output. The list of publications valid for research funding until 2010 was based on somewhat ill-defined criteria but the Ministry has updated and revised the list successively in September and December 2012 and is available on the homepage of the Ministry: http://www.nauka.gov.pl/finansowanie/finansowanie-nauki/dzialalnosc-statutowa/. The list of publications is composed of three parts. Part A includes journals, irrespective of their language, belonging to Thomson Reuters’ journal IFs and are found in the *Journal Citation Reports* (JCR) database. A paper published in these journals has been assigned points lying between 15 and 50 (in steps of 5 points), depending on the journal IF. Journals in Part B are those which do not have IFs, and a paper published in these journals is assigned between 1 and 9 points (in steps of 1 point). Journals in Part C, on the other hand, are from the *European Reference Index for the Humanities* database, and a paper in these journals is assigned 10, 12 and 14 points. A cursory examination of these lists of journals reveals that several journals from the previous list B have entered the new list A of IF journals and some of them have even IF exceeding unity. This is a result of inclusion of more and more national journals in the Thomson Reuters databases in recent years.

For the evaluation of research performance most funding agencies usually use citation data from journals for disciplines such as Science, Engineering and Medicine from Thomson Reuters’ Web of Science (WoS). They do not use citation data for disciplines such as Social Sciences and Humanities from WoS or Scopus databases because these databases do not cover citations in books, book chapters, conference papers or journals not indexed in the WoS. Google Scholar has been reported to represent poor coverage for disciplines such as Chemistry and Physics and has a wide coverage which does not vary much across different fields and often includes nearly 90 % of published outputs including books and reports (Harzing 2013; Mingers and Lipitakis 2010). However, the citations it generates come from many different sources which are often not research related (Mingers and Lipitakis 2010). Mingers and Lipitakis (2010) reported that in the field of business and management WoS is more accurate and rigorous. In a recent study, Harzing (2013) compared of coverages of the publication output of 20 Nobel Prize winners in Chemistry, Economics, Medicine and Physics by Google Scholar and WoS, and found that: (1) Google Scholar might provide a less biased comparison across disciplines than the WoS and (2) the use of Google Scholar might redress the traditionally disadvantaged position of the Social Sciences in citation analysis.

The IF of a journal in a particular year is defined as the ratio of the number of citations received in that year by papers published in the journal in the previous 2 years to the number of papers published in that journal in those 2 years. Since it is a measure of the mean citations per paper over a two-year period, there are a number of problems associated with this measure, which are mainly concerned with the short time window for citation record, the robustness/reliability of data sources, and the coverage of data by the source. These problems of the journal IF have been accentuated over years in the literature. The problems are essentially directed to Thomson Reuters which manages its “World of Science” databases used for the calculation of IF of journals. To address the criticism of two-year impact factors (IF2s) of journals, Thomson Reuters has taken a number of steps. For example, since 2007 World of Science database has started publishing five-year impact factors (IF5s) of journals in addition to their classical two-year impact factors (IF2s), and during the last 5 years Thomson Reuters has successively expanded its databases by including new English, non-English and multilingual journals published in different countries across the World.

According to Zitt (2012) the limitations of IF are not its flaw as a measure but it is the vulnerability of the measure to changes, including manipulation, by issues such as the type and the number of documents fetching citations. For example, impact factors of journals can be increased by including high number of self-citations (Bornmann et al. 2008), because journal self-citations are included in the calculation of impact factors. However, despite recognized deficiencies of impact factors of journals, their adoption as a measure of scientific performance has resulted in an omnipresent pressure on editors to improve the impact factors of their journals and on authors to publish in journals with high impact factors, .

Didegah et al. (2012) compared journal publishing behaviors against journal citing behaviors across the world. These authors found that: (1) most papers in five ranges of percentiles of IF2-based quality, from the top 1 %, followed by 1–10 %, 10–20 % and 20–50 %, to the lowest 50–100 %, of journals come from scientifically and economically advanced countries, (2) less developed countries cite high-quality journals at the same rate as developed countries, and (3) research cooperation between developed and less developed countries positively influences the publishing behavior of the latter as their papers coauthored with developed countries are published more often in top quality journals. The influence of research collaboration between countries on their citation impact is also well known. For a review on this subject the reader is referred to a recent paper by Lancho-Barrantes et al. (2013).

Guerrero-Bote et al. (2007) suggested that the distribution of IF of journals belonging to a particular subject category on the journal rank is related to rates of export and import of knowledge in a subject area, denoted here by EX and IM, respectively, defined by the following relations:

1 $$ EX = \frac{{L_{\text{total}} - L_{\text{sc}} }}{{L_{\text{total}} }}, $$

2 $$ IM = \frac{{R_{\text{total}} - L_{\text{sc}} }}{{R_{\text{total}} }}, $$

where *L*_total_ is the number of all citations received in the year Y by papers published in the year (Y-3), (Y-2) or (Y-1) in a subject category, *L*_sc_ is the number of subcitations (citations from journals of the same subject area) received by the above papers in the year Y, and *R*_total_ is the number of references of the category. The concept of export and import rates of knowledge, called the iceberg hypothesis, was also explored in a later paper by Lancho-Barrantes et al. (2010) to describe the rank-order distribution of IF in several other subject categories. According to the present author, the above EX and IM parameters do not give important information on the citation behavior of journals belonging to a subject category. For example, the difference (*L*_total _− *L*_sc_) is equal to the number of citations from journals not from the same subject area and is directly connected to the “external impact” factor (IF_ext_) defined by the above authors such that IF_ext_ < IF. In fact, one observes IF > IF_ext_ in the plots of IF of journals belonging to various subject categories against the descending journal rank, reported in the above papers. However, apart from the iceberg hypothesis, various other mathematical functions have been proposed to describe the rank-order distributions of items, including IF of journals in various scientific disciplines. For a brief survey of the literature on this subject the reader is referred to a recent paper by the present author (Sangwal 2013).

There is sparse literature on the study of the comparative behavior of journals published in individual countries in English and national languages. No special attention has also been paid until now to analyze the influence of self-citations of journals published in different countries on their impact factors. The present study is addressed to these issues using Thomson Reuters’ JCR databases. The aim of the study is three-fold: (1) to compare citation-related characteristics of journals published in nine individual countries from an analysis of their publication languages, two-year and five-year impact factors and self-citations, (2) to examine the factors which lead to changes in the impact factors, and (3) to analyze self-citation characteristics of journals in terms of their publication languages.

## Bibliometric data for analysis

We analyzed the citation data of journals published in the following nine countries: Brazil, Croatia, Czech Republic, Italy, Poland, Romania, Slovakia, Spain and Turkey. The countries were selected from the consideration that English is not the national language of these countries, and a high percentage of journals are published in their national languages in different scientific disciplines. Due to their geographical, political and economic background, they represent different publication cultures and organization of research work. For example, in Czech Republic, Poland, Slovakia and Romania research work is carried out in universities as well as institutes of their national academies of sciences but practically in all of the countries considered in this study there are independent research institutes.

We used JCR of Thomson Reuters ISI Web of Knowledge database covering the period 2002–2011, to collect appropriate bibliometric data about the journals, their publication language, publishers, two-year impact factors with journal self-citations (IF2) and without self-citations (IF2_nsc_), five-year impact factors with self-citations (IF5), journal subject category quartiles (Q1–Q4) based on quartiles of categories, and journal self-citations from the above selected countries. Some basic information about the journals from the 2008–2012 JCR Science Edition is collected in Tables 1 and 2. Table 2 contains data on the numbers of journals from the investigated countries in the 2008 and 2011 JCR databases in: (1) subject category quartiles Q1, Q2, Q3 and Q4, from the topmost subject category Q1 to the lowest subject category Q4, assigned according to the distribution of their decreasing IF2 in the percentile ranges 100–75 %, 75–50 %, 50–25 % and 0–25 %, respectively, in different scientific areas, (2) journal self-citation quartiles F1, F2, F3 and F4 from the lowest to the highest self-citations, assigned according to the distribution of their increasing self-citations, in the percentile ranges 0–25 %, 25–50 %, 50–75 % and 75–100 %, respectively, and (3) English-language (Engl.), multilingual (ML) and national-language (N) journals. Data on subject category quartiles Qs are given in the JCR databases, whereas ranges of journal self-citation quartiles Fs were calculated from the values of ratio *f* = IF2_nsc_/IF2 (see Fig. 7) or from self-citations percentiles. The values of IF2, IF2nsc and percentage self-citation of different journals are given in the JCR databases.

**Table 1:** Total number *N* and number *N*_IF5_ of journals with IF5 from different countries indexed in JCR of 2008–2011

Country	*N* (*N*_IF5_) in different years			
	2008	2009	2010	2011
Poland	59 (51)	103 (52)	122 (56)	126 (55)
Italy	75 (59)	100 (64)	121 (69)	125 (78)
Brazil	28 (18)	65 (22)	89 (30)	96 (32)
Spain	37 (29)	60 (34)	73 (37)	78 (39)
Romania	10 (8)	33 (8)	44 (9)	47 (9)
Turkey	8 (3)	32 (7)	49 (12)	54 (12)
Croatia	11 (11)	24 (12)	35 (12)	36 (11)
Czech Republic	22 (20)	31 (20)	32 (21)	33 (23)
Slovakia	11 (10)	16 (9)	19 (11)	19 (12)

**Table 2:** Research categories, publication languages and self-citations of journals published in selected countries

Country	JCR	Quartile in category					Language			Quartile in journal self-cites				
		Q1	Q2	Q3	Q4	ΣQ	Engl.	ML	Local	F1	F2	F3	F4	ΣF
Poland	2008	0	8	21	42	71	46	12	1	45	12	2	0	59
	2011	0	18	45	88	151	87	17	22	66	31	17	12	126
Italy	2008	3	16	26	59	104	52	19	3	61	10	3	0	74
	2011	17	19	39	84	159	87	19	17	88	27	8	1	124
Brazil	2008	0	5	12	19	36	12	12	4	18	6	3	1	28
	2011	0	8	25	78	111	33	18	45	55	28	12	1	96
Spain	2008	1	9	18	19	47	15	11	11	22	8	7	0	37
	2011	2	11	20	62	95	15	24	37	43	23	8	2	76
Turkey	2008	0	0	3	6	9	5	2	1	5	2	1	0	8
	2011	3	2	12	48	65	24	6	24	27	16	10	1	54
Romania	2008	3	0	3	10	16	4	3	3	4	1	3	2	10
	2011	2	6	15	28	51	33	4	10	17	10	14	6	47
Croatia	2008	0	1	4	7	12	5	5	1	10	0	1	0	11
	2011	0	2	13	29	44	23	7	6	21	9	5	1	36
Czech Rep.	2008	1	2	12	11	26	12	10	–	13	5	3	1	22
	2011	0	7	16	15	38	23	10	–	19	12	1	1	33
Slovakia	2008	0	0	4	10	14	9	2	–	10	1	0	0	11
	2011	1	0	4	19	24	16	3	–	14	5	0	0	19

It should be mentioned that all non-English journals published in Czech Republic and Slovakia publish papers both in Czech and Slovak languages in addition to papers in English. Therefore, journals published in Czech Republic and Slovakia are typically either English-language or multilingual.

## Two-year versus five-year impact factors of journals

All of the journals indexed in the 2008–2011 JCR databases do not have their two-year impact factors (IF2). This situation is observed, for example, in the case of Spain for 2011 journals, where 2 journals do not have their IF2. However, not all of the journals with IF2 have their five-year impact factors (IF5) and the number *N*_IF2_ of journals with IF2 is usually much higher than the number *N*_IF5_ of journals with IF5. This difference is due to the inclusion in the successive JCR databases of new journals which did not have citation data covering five-year window.

We examined the influence of duration of citation window on impact factors of journals by investigating the relationship between two-year IFs (IF2) of journals published in different countries and their corresponding five-year IFs (IF5). For this purpose we selected the 2011 JCR database which has indexed the highest number of journals among the four databases analyzed here.

**Fig. 1:**
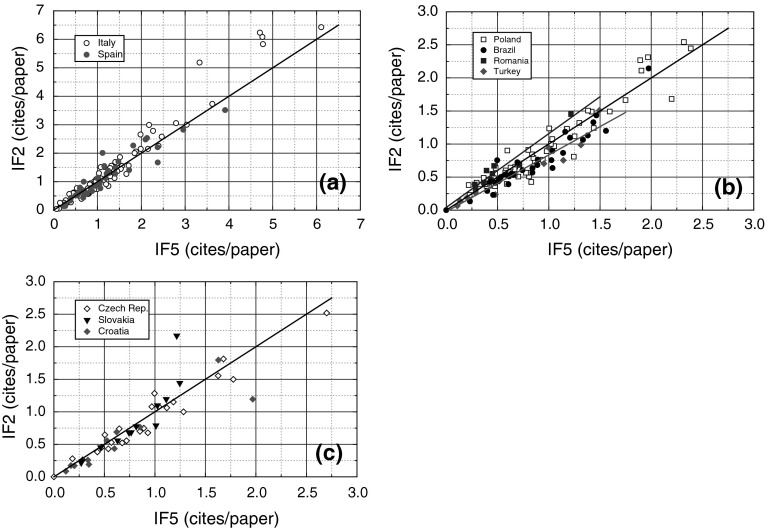
Plots of IF2 of journals published in different countries against their corresponding IF5 according to 2011 JCR. *Linear plot* represents a *slope* of unity. For the sake of clarity data are presented in separate figures

Figure 1 shows the dependence of the values of IF2 of journals published in different countries on their corresponding IF5, whereas the solid linear plots represent a slope of unity when IF2 = IF5 for different journals. The slope of the plots of IF2 against IF5 of different journals published in Spain (Fig. 1a), Poland (Fig. 1b), Croatia, Czech Republic and Slovakia (Fig. 1c) is approximately unity. In contrast to these cases of the slope of unity, for the journals published from Turkey and Brazil the slope is lower than unity whereas that for the journal from Romania exceeds unity, as indicated by the dashed line in Fig. 1b. Since the journal impact factor is computed as the ratio of citations received in a given year by papers published over a citation window, these features of the plots of IF2 against IF5 with slopes of lower than, equal to or higher than unity are related to the general trends of increasing, constant or decreasing number of citations received in successive years by the journals published in these countries, respectively. The values of IF2 higher than those of IF5 for the journals published by a country mean higher values of citations during the 2 years of citations considered in the calculations of IF2, whereas lower values of IF2 imply that the journals received lower citations during the 2 years.

It is interesting to confront the above general conclusions drawn from a comparison of the IF2 and IF5 of high-ranked international journals and top-ranked journals published from another country. Raj and Zainab (2012) recently reported, among others, data on the IF2 and IF5 of top ten international journals from Thomson Reuters 2008 JCR database and of top ten national journals from the Malaysian citation database. Examination of the data for Malaysian journals reveals that for most of these journals their IF2 is higher than IF5 and for two journal this increase is even 170 and 260 %. Obviously, the citation behavior of Malaysian journals is somewhat similar to that of Romanian journals. However, in the case of data on the international journals, except in the case of one journal (CA-Cancer J. Clin.) where the IF2 (74.58) has increased substantially from its IF5 (50.77), the values of IF2 of the remaining journals have either remained practically constant or somewhat decreased (by <20 %). The present author also examined the recent data of IF2 against IF5 for the top 20 international journals from 2011 JCR database. It was found that, with the exception of the journal CA-Cancer J. Clin. (IF2 101.78; IF5 67.41) where IF2 differs from IF5 enormously, IF2 has remained comparable with IF5 for most of the journals.

Campanario (2011) compared the values of IF2 with those of IF5 of top 20 international journals from Thomson Reuters 2007–2009 JCR databases and found that IF5 > IF2 for most journals but IF5 < IF2 for about a quarter of them. Similar observations have previously been made by other authors (Rousseau et al. 2001). The increase in IF2 of journals was attributed to the citations of more papers published in the latest 2 years than in the previous years (Campanario 2011). Using the scientific publication output of Norwey, Aksnes and Sivertsen (2004) found that: (1) there are large annual variations in the influence of highly cited papers on the average citation rate of the subfields, and (2) the average citation rates of papers in major subfields are highly determined by one or only a few highly cited papers. The above observations are associated with the highly skewed distribution of citations of papers published in journals. Therefore, IF is increased primarily by the highly cited papers (Vinkler 2012; Moed et al. 2012). In view of this skewness of citation distribution of papers in journals, a huge number of citations received by an individual paper published in a journal can have a dramatic effect on its IF (Moed et al. 2012).

## Publishing trends of journals

The growth dynamics of the journals published by different countries may be analyzed from the dependence of the ratio (*N*_IF2 _− *N*_IF5_)/*N*_IF2_ on the number *N*_IF2_ of journals with IF2. The interval in the values of (*N*_IF2 _− *N*_IF5_)/*N*_IF2_ in the plots of (*N*_IF2 _− *N*_IF5_)/*N*_IF2_ against *N*_IF2_ for different countries is a measure of “established” journals published in different countries. The lower and narrower the interval in the values of (*N*_IF2 _− *N*_IF5_)/*N*_IF2_ for a country, the higher is the number of the established journals published by it. However, the slope of the plot of (*N*_IF2 _− *N*_IF5_)/*N*_IF2_ against *N*_IF2_ is a measure of the growth dynamics of the journal published in different countries. The lower the value of the slope of the plot of (*N*_IF2 _− *N*_IF5_)/*N*_IF2_ as a function of *N*_IF2_ for a country, the higher is the growth dynamics of the journals published in it.

**Fig. 2:**
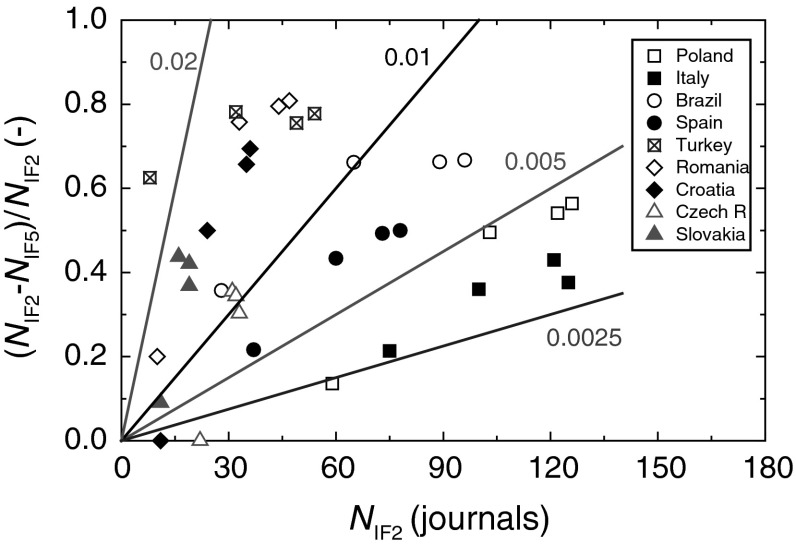
Plots of (*N*_IF2 _− *N*_IF5_)/*N*_IF2_ against the total number *N*_IF2_ of journals with IF2 published in different countries. Data from Table 1

Figure 2 shows the plots of (*N*_IF2 _− *N*_IF5_)/*N*_IF2_ against *N*_IF2_ for different countries. Four linear plots of (*N*_IF2 _− *N*_IF5_)/*N*_IF2_ on *N*_IF2_ drawn with slopes of 0.02, 0.01, 0.005 and 0.0025 are also shown in the figure for visual reference. The values of the slope of the plots of (*N*_IF2 _− *N*_IF5_)/*N*_IF2_ against *N*_IF2_ for different countries indicate that the highest growth dynamics of journals has occurred in countries like Italy and Turkey, whereas the lowest growth dynamics has been observed by journals published in Croatia, Czech Republic and Slovakia. The growth dynamics of journals published in Spain, Brazil, Poland and Romania lies in between the above two extremes.

The total number *N* of journals published in the countries considered here for 2008 as well as 2011 JCR databases is always lower than the total number *N*_Q_ of subject categories represented by them. For example, according to the 2011 JCR database, the total number of journals published by the countries analyzed in this study is 610 but they are assigned to 738 subject categories. This is due to the fact that many journals are assigned to more than one JCR category. For example, *Opto*-*Electronic Review* (Opto-Electron. Rev.), published in Poland by Versita, a Publisher with publication/distribution arrangements with Springer, and *Energy Education Science and Technology* (Energy Educ. Sci. Tech.), published in Turkey by Sila Science (University Mah, Trabzon), belong to three categories. The share of more than one JCR category in the journals, defined here as percentage of excess of categories (excess %), lies in a wide range for these countries. This excess share lies between 8.5 and 29.3 % for the journals indexed in the 2011 JCR database. The lower the value of the excess share of categories, the higher is the percentage of one-category journals. The 2011 JCR data reveal that a large proportion of journals published in Romania belongs to one-category journals, a large proportion of journals published in Croatia, Czech Republic, Slovakia and Spain are two-category journals, whereas the journals published in Brazil, Italy, Poland and Turkey belong to two as well as three subject categories.

**Fig. 3:**
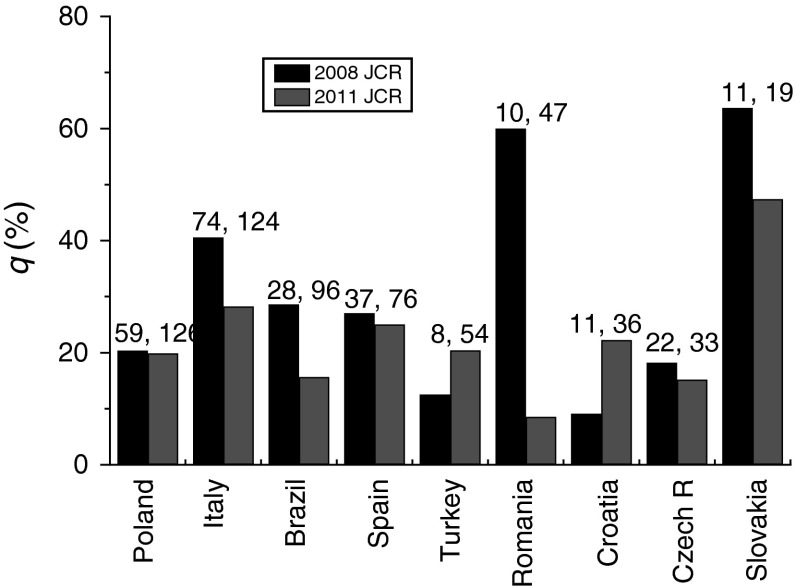
Dependence of parameter *q* = (*N*_Q _− *N*_IF2_)/*N*_IF2_ on the number *N*_IF2_ of journals published in different countries. *N*_Q_ is the total number of categories. Data from Table 2. See text for details

The behavior of excess subject categories for journals published in the countries analyzed here in different years was compared by introducing the parameter *q* = (*N*_Q _− *N*_IF2_)/*N*_IF2_, where *N*_Q_ is the total number of categories and *N*_IF2_ is the number of journals with IF2. We used *N*_IF2_ values of journals instead of the number *N* of indexed journals because IF2 of a journal is used to assign a category to it. The values of the parameter *q* for journals indexed in 2008 and 2011 for different countries are compared in Fig. 3. The number *N*_IF2_ of journals with IF2 for these 2 years are given at the top of the two columns for each country.

It may be seen from the figure that the parameter *q* is not directly related to the number *N*_IF2_ of journals published in a country. However, with increasing number *N*_IF2_ of journals published by individual countries, the values of *q* show enormously different trends. With an increase in *N*_IF2_, the value of *q* for Poland, Spain and Czech Republic remains essentially unchanged, it decreases for Romania, Brazil, Italy, and Slovakia, whereas it increases for Turkey and Croatia. These observations are related to changes in the ratio *N*_Q_/*N*_IF2_ with an increase in the number *N*_IF2_ (i.e. *N*) of journals published in a country. When more new journals from a country with a smaller number of subject categories than those in previous years are indexed in the JCR database, the value of *q* decreases in later years. When more new journals from a country with a higher number of subject categories than those in previous years are included in the JCR database, the value of *q* increases in subsequent years. However, when more new journals from countries with the same number of subject categories as in previous years are included in the JCR database, *q* remains unchanged in later years.

## Journal categories and self-citation

**Fig. 4:**
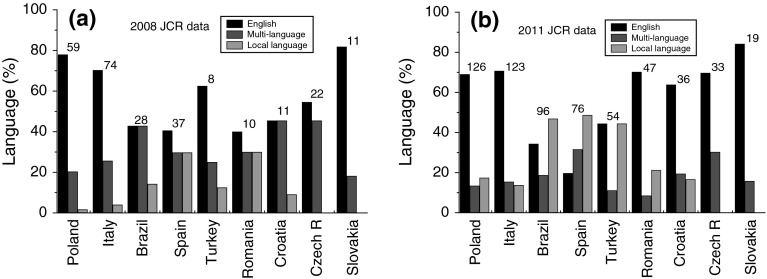
Histogram of relative participation of English, multi-language and local language journals published in different countries according to **a** 2008 JCR and **b** 2011 JCR. Total number of journals from a country is given at the *top* of corresponding *columns*. Data from Table 2

Figure 4 compares the relative percentages of English-language, multilingual and national-language journals published in different countries according to 2008 and 2012 JCR databases. Several features may be noted from this figure:

1. In the 2008 JCR data the share of English-language journals is always higher than that of national-language journals. However, the share of national-language journals is relatively high about 30 % in countries like Spain and Romania and is equal to that of multilingual journals. In contrast to this, the share of English-language and multilingual journals is equal and is about 45 % for Brazil and Croatia.
2. The share of English-language journals has remained practically at the same level in the 2008 and 2011 JCR databases for Poland, Italy and Slovakia. However, in 2011 JCR database the relative share of English-language journals published in Romania has practically doubled with respect to the 2008 JCR database at the expense of Romanian-language and multilingual journals.
3. In the case of Czech Republic and Slovakia publishing English-language and multilingual journals alone, their relative shares in the 2008 and 2011 JCR databases follow different trends. The relative shares of the English-language and multilingual journals from Slovakia have remained practically unchanged at about 85 and 15 %, respectively, but the share of English-language journals published in Czech Republic has increased significantly in the 2011 JCR database at the expense of multilingual journals.
4. The total number of national-language journals indexed in the 2011 JCR database for all countries has increased to 161 from mere 24 indexed in the 2008 JCR database. This share has approached 26.4 % of the total number of journals in the 2011 JCR database from 9.4 % of the journals in the 2008 JCR database.

**Fig. 5:**
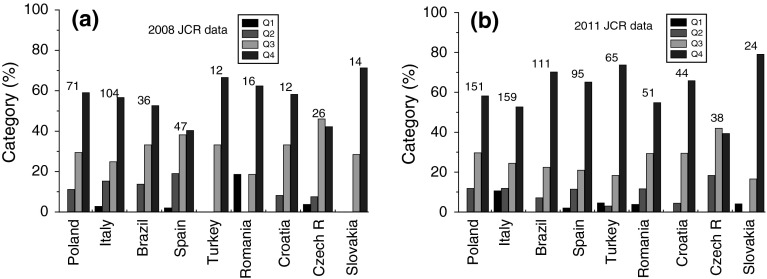
Histogram of relative participation of four quartiles in the subject categories of journals published in different countries according to **a** 2008 JCR and **b** 2011 JCR. Total number of categories for journals from a country is given at the *top* of corresponding *columns*. Data from Table 2

Figure 5 shows the relative distribution of four quartiles of the subject categories of journals published in different countries according to 2008 and 2011 JCR databases. As seen from Fig. 5, with insignificant changes in the order of neighboring categories, the share of journals published in a country increases with lowering of their category in the two databases. Among these insignificant changes are an increase or a decrease in categories Q1 and Q2 for different countries, but one also encounters redistribution of shares of categories Q1 and Q2 for a country in the two databases. Large changes are observed in the case of Italy, Spain, Turkey and Romania. The shares of Q1 and Q2 have increased for Italy and Turkey, whereas the shares of different categories have become steadily increasing for Spain and Romania in the 2011 JCR database in comparison with those in the 2008 database.

**Fig. 6:**
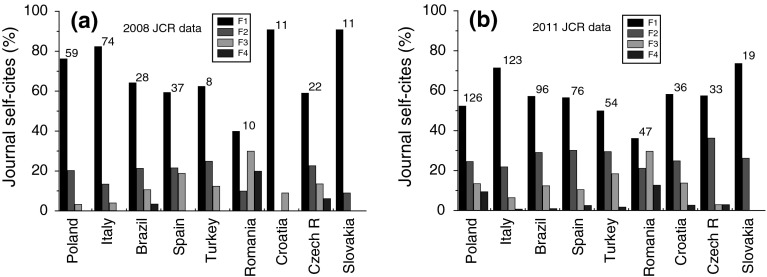
Histogram of relative participation of four quartiles in the groups of self-citations to journal published in different countries according to **a** 2008 JCR and **b** 2011 JCR. Total number of journals from a country is given at the *top* of corresponding *columns*. Data from Tables: **a** 3 and **b** 4

Figure 6 shows the relative distribution of self-citation quartiles F of journals published in different countries according to 2008 and 2011 JCR databases. It may be noted that, with the exception of Romania, the relative distribution of self-citation quartiles of journals published in various countries decreases with increasing journal self-citations. However, there are relatively high shares of self-citations quartiles F3 and F4 in the case Romania.

The effect of self-citations and publication languages of journals published in different countries was examined from the distribution of categories of English and non-English journals corresponding to different journal self-citations quartiles. The relevant data are given in Tables 3 and 4 according to the 2008 and 2012 JCR databases. From these tables the following features may be noted:

**Table 3:** Structure of quartiles of categories Q of English and non-English journals from 2008 JCR database corresponding to different quartiles of journal self-citations

Country	Self-cite Quartile	English journals				Non-English journals			
		Q1	Q2	Q3	Q4	Q1	Q2	Q3	Q4
Poland	F1	0	4	14	28	0	0	1	0
	F2	0	3	2	4				
	F3	0	0	1	0				
Italy	F1	2	12	16	33	0	0	0	2
	F2	1	2	3	2	0	0	0	0
	F3	0	0	1	3	0	0	0	2
Brazil	F1	0	2	6	4	0	1	1	3
	F2	0	0	0	1				
	F3	0	0	0	3				
Spain	F1	1	4	6	6	0	0	2	3
	F2	0	1	2	0	0	0	0	3
	F3					0	2	3	1
	F4								
Turkey	F1	0	0	2	3				
	F2	0	0	1	0				
	F3					0	0	0	1
Romania	F1	3	0	0	0				
	F2	0	0	1	2	0	0	0	1
	F3	0	0	0	2	0	0	0	2
	F4	0	0	0	1	0	0	1	0
Croatia	F1	0	1	3	3	0	0	0	1
Czech Rep.	F1	0	1	4	4				
	F2	1	0	1	2				
	F3	0	0	1	1				
Slovakia	F1	0	0	2	9				
	F2	0	0	1	0

**Table 4:** Structure of quartiles of categories Q of English and non-English journals from 2011 JCR database corresponding to different quartiles of journal self-citations

Country	Quartile in journal self-cites	English journals				Non-English journals			
		Q1	Q2	Q3	Q4	Q1	Q2	Q3	Q4
Poland	F1	0	6	24	37	0	0	0	5
	F2	0	5	9	11	0	0	0	7
	F3	0	3	3	7	0	0	1	4
	F4	0	0	3	1	0	1	0	5
Italy	F1	9	9	27	42	0	0	1	9
	F2	6	5	6	9	0	0	0	4
	F3	0	0	0	2	0	0	0	2
	F4	0	0	0	2	0	0	0	2
Brazil	F1	0	5	7	13	0	0	1	22
	F2	0	0	2	8	0	2	4	15
	F3	0	1	0	1	0	0	1	8
	F4					0	0	0	1
Spain	F1	0	6	4	6	0	0	2	14
	F2	0	2	2	1	1	0	3	14
	F3					0	0	0	9
	F4					0	1	0	1
Turkey	F1	0	1	7	12	0	0	0	12
	F2	0	0	1	5	0	0	1	6
	F3	0	1	1	2	0	0	1	4
	F4	3	0	0	0				
Romania	F1	2	2	5	10				
	F2	0	1	1	3	0	0	1	1
	F3	0	1	4	4	0	0	1	4
	F4	0	0	1	2	0	0	1	2
Croatia	F1	0	2	7	9				
	F2	0	0	0	7	0	0	1	2
	F3	0	0	0	3	0	0	0	1
	F4					0	0	0	1
Czech Rep.	F1	0	3	5	7				
	F2	0	2	6	3				
	F3	0	1	0	1				
Slovakia	F1	1	0	1	14				
	F2	0	0	1	3

1. The number of subject category quartiles Q of English-language journals published in a country is mainly confined to self-citation quartiles F1 and F2. However, a majority of the journals in these self-citation quartiles lies in subject categories Q2, Q3 and Q4. Journals published in Romania are exceptions.
2. The number of subject category quartiles Q of English-language journals published in a country increases in the case of self-citation quartile F1 of journal, but no specific trend of the number of categories is observed for other self-citation quartiles of journals published in different countries.
3. In Brazil, Spain and Turkey, where the percentage of non-English-language journals is comparable with or higher than that in the case of English-language journals, a majority of the journals belongs to self-citation quartiles F1 and F2 but most of them lie in subject categories Q3 and Q4. In contrast to these countries, in Romania there are no journals belonging to self-citation quartile F1. However, most of the non-English journals published in all countries belong to subject category Q4.

From the above observations it may be concluded that the subject categories of non-English-language journals published in different countries follow trends different from those in the case of English-language journals. Non-English-language journals mainly belong to the lowest category Q4 in comparison with English-language journals a majority of which belongs to categories Q3 and Q4. In other words, English-language journals have higher impact factors than non-English journals. This inference is consistent with the previous findings on differences in the citations of English- and non-English-language journals (Garfield 1978; Gonzalez-Alcaide et al. 2012; Liang et al. 2013; Mueller et al. 2006; Poomkottayil et al. 2011; Sangwal 2012; van Raan et al. 2011).

There are no non-English-language journals published in Croatia and Romania belonging to self-citation quartile F1, whereas there are comparable but relatively high percentage of category Q4 journals published in these countries belonging to self-citation quartiles F3 and F4. This trend of the percentage of category Q4 journals belonging to self-citation quartiles F3 and F4 is different from that encountered in the case of non-English-language journals published in the other countries, and is associated with relatively high contribution of self-citations in the case of Croatia and Romania.

Examination of subject areas of the journals published in different countries revealed that in practically all countries non-English-language journals cover highly specialized areas like agriculture, horticulture, forestry, agronomy, food sciences and technology, veterinary sciences, fisheries, nursing, surgery, oncology, dermatology, cardiology, pediatrics and general and internal medicine. Similar findings have been reported earlier in the case of Spanish-language journals in the fields of clinical medicine or social sciences and humanities (Gonzalez-Alcaide et al. 2012). The main reason of this trend is associated with the localized nature of the subject matter of the papers published in non-English-language journals. Therefore, these journals are not attractive for a relatively wide range of audience, especially publishing their papers in English-language journals. This results in poor citations of the papers published in non-English-language journals and their low impact factors. Consequently, these journals are expected to belong to relatively low category quartiles in comparison with English-language journals.

## Self-citation characteristics of English- and non-English-language journals

From the nine countries selected above, the bibliometric data for the journals published in the seven countries (i.e. Brazil, Croatia, Italy, Poland, Romania, Spain and Turkey), containing papers written in English, in the national language of a country, or in both of these languages, were analyzed in detail for 2008 and 2011. Journals published in the remaining two countries, Czech Republic and Slovakia, which contain papers written in English alone or in both Czech and Slovak languages in addition to papers in English, were not considered for the analysis in view of relatively small data and absence of typically national-language journals.

**Fig. 7:**
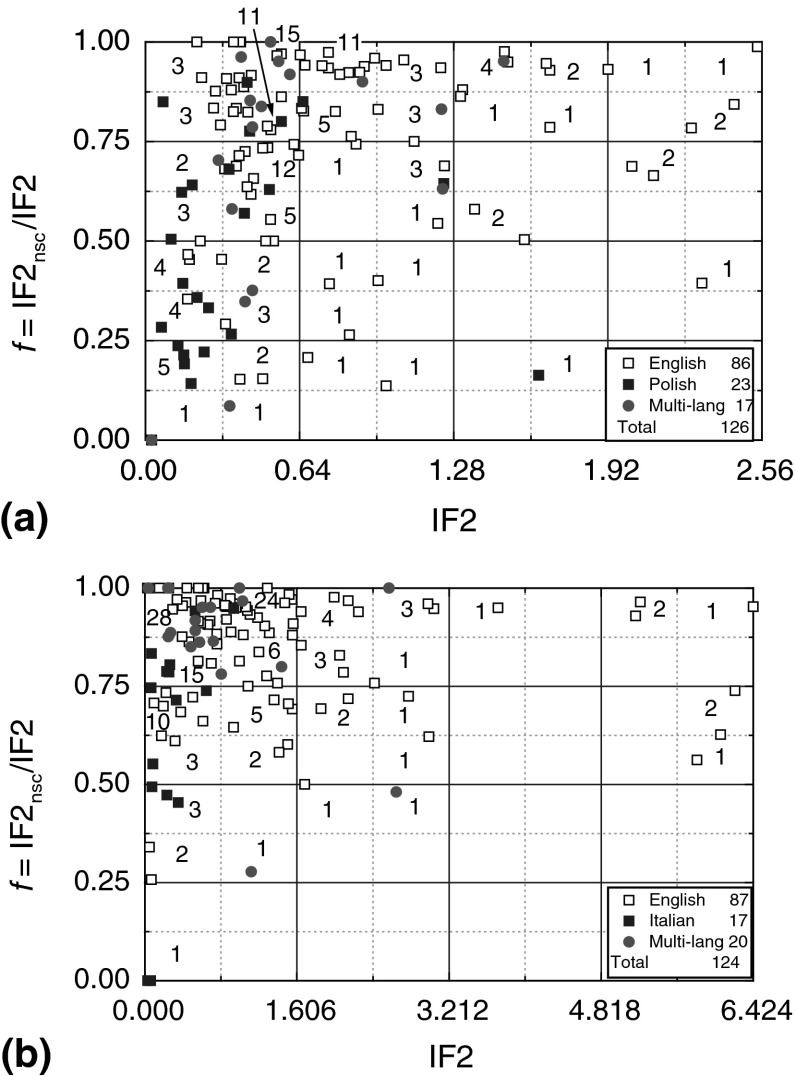
Typical examples of relationship between parameter *f* = IF2_nsc_/IF2 and IF2 of self-citations of English-, national- and multi-language journals published in **a** Poland and **b** Italy. Original data from 2011 JCR database. See text for details

In order to investigate the influence of publication language of journals, the distribution of English-, national- and multi-language journals published in different countries was analyzed quantitatively from the number *N*_E_, *N*_N_ and *N*_ML_ of journals, respectively, in self-citation quartiles F1–F4. The numbers *N*_E_, *N*_N_ and *N*_ML_ of journals in the self-citation quartiles F1–F4 may be counted in two ways: (1) directly from the printouts of datafiles, with additional information recorded manually of the values of percent self-citations or of two-year impact factors without self-citations (IF2_nsc_), from databases for the journals from different countries or (2) from the plots of self-citation parameter *f*, calculated from the values of IF2_nsc_ and two-year impact factors with self-citations (IF2) as *f* = IF2_nsc_/IF2, as a function of IF2 of journals published in a country. Figure 7 shows typical examples of the plots of journal self-citation parameters *f* for the English-, national- and multi-language journals published in different countries plotted as a function of the values of their IF2 from the 2011 JCR database.

It should be mentioned that the definition of the parameter *f* introduced above is similar to that of the parameter EX of Eq. (1), used by Guerrero-Bote et al. (2007). The definition of *f* is based on IF2 and IF2_nsc_ which correspond to the citations *L*_total_ and *L*_nsc_ normalized with respect to the number *N*_IF2_ of papers with IF2 published in a given period. For a given year when *N*_IF2_ is constant, *f* = EX. However, for different years when *N*_IF2_ does not remain constant and the citation behavior of journals from a country is also different, *f* ≠ EX.

In Fig. 7, IF2s of the journals from a country are grouped into four quartiles defined by the parameter *g* = IF2/IF2_max_, where IF2_max_ is the highest IF2 for a country. An exception is the IF2 = 31.677 of the top journal from Turkey, where the highest IF2 is taken as 2 which is approximately equal to the second top journal with IF2 = 1.991. The groups G1, G1, G3 and G4 defined in this way on the basis of IF2 quartiles are: (1) 0–0.25, (2) 0.25–0.50, (3) 0.5–0.75 and (4) 0.75–1. This categorization of IF2 into G groups is similar to that of categorization of IF2 into subject category quartiles Q used in Thomson Reuters JCR databases. Each self-citation quartile F and each IF2-based group quartile G were further divided into two subgroups. The numbers of journals located in these different subgroups of self-citation and IF2-based group quartiles are denoted in these figures whereas for different countries the numbers of English-, national- and multi-language journals and their total number published are given in the insets.

The numbers *N*_E_, *N*_N_ and *N*_ML_ of parameter *f* corresponding to groups F1–F4 for English-, national- and multi-language journals, counted by following the above procedure, from various countries are given in Tables 5 and 6 for 2008 and 2011 JCR databases, respectively. As noted before from Table 2, these tables also show that most of the journals published in different countries belong to self-citation groups F1 and F2 but there are several exceptions where a high percentage of journals belongs to self-citation groups F3 and F4.

**Table 5:** Numbers *N* of journals with different quartiles F of self-citations according to 2008 JCR database

Countries	Self-cite quartile	Number of journals^a^			
		*N*_E_	*N*_N_	*N*_ML_	Total
Poland	F1	36	1	8	37
	F2	9	–	3	9
	F3	1	–	1	1
	Sum	46	1	12	59
Italy	F1	43	1	17	61
	F2	7	1	2	10
	F3	2	1	–	3
	Sum	52	3	19	74
Brazil	F1	9	1	8	18
	F2	1	3	2	6
	F3	2	0	1	3
	F4	0	0	1	1
	Sum	12	4	12	28
Spain	F1	13	3	6	22
	F2	2	3	3	8
	F3	0	5	2	7
	Sum	15	11	11	37
Turkey	F1	4	0	1	5
	F2	1	0	1	2
	F3	0	1	0	1
	Sum	5	1	2	8
Romania	F1	1	0	3	4
	F2	1	0	0	1
	F3	1	2	0	3
	F4	1	1	0	2
	Sum	4	3	3	10
Croatia	F1	5	1	4	10
	F3	0	0	1	1
	Sum	5	1	5	11

^a^Lower indexes E, N and ML denote English, national and multi-language journals

**Table 6:** Numbers *N* of journals with different quartiles F of self-citations according to 2011 JCR

Countries	Self-cite quartile	Number of journals^a^			
		*N*_E_	*N*_N_	*N*_ML_	Total
Poland	F1	51	5	10	66
	F2	21	7	3	31
	F3	10	5	2	17
	F4	4	6	2	12
	Sum	86	23	17	126
Italy	F1	66	6	16	88
	F2	23	4	0	27
	F3	3	3	2	8
	F4	0	1	0	1
	Sum	92	14	18	124
Brazil	F1	23	18	14	55
	F2	6	19	3	28
	F3	3	8	1	12
	F4	1	0	0	1
	Sum	33	45	18	96
Spain	F1	20	10	13	43
	F2	1	17	5	23
	F3	0	6	2	8
	F4	0	1	1	2
	Sum	21	34	21	76
Turkey	F1	14	11	2	27
	F2	5	7	4	16
	F3	4	6	0	10
	F4	1	0	0	1
	Sum	24	24	6	54
Romania	F1	15	0	2	17
	F2	8	1	1	10
	F3	9	5	0	14
	F4	2	3	1	6
	Sum	34	9	4	47
Croatia	F1	15	1	5	21
	F2	5	3	1	9
	F3	3	1	1	5
	F4	0	1	1	1
	Sum	23	6	7	36

^a^Lower indexes E, N and ML denote English, national and multi-language journals

**Fig. 8:**
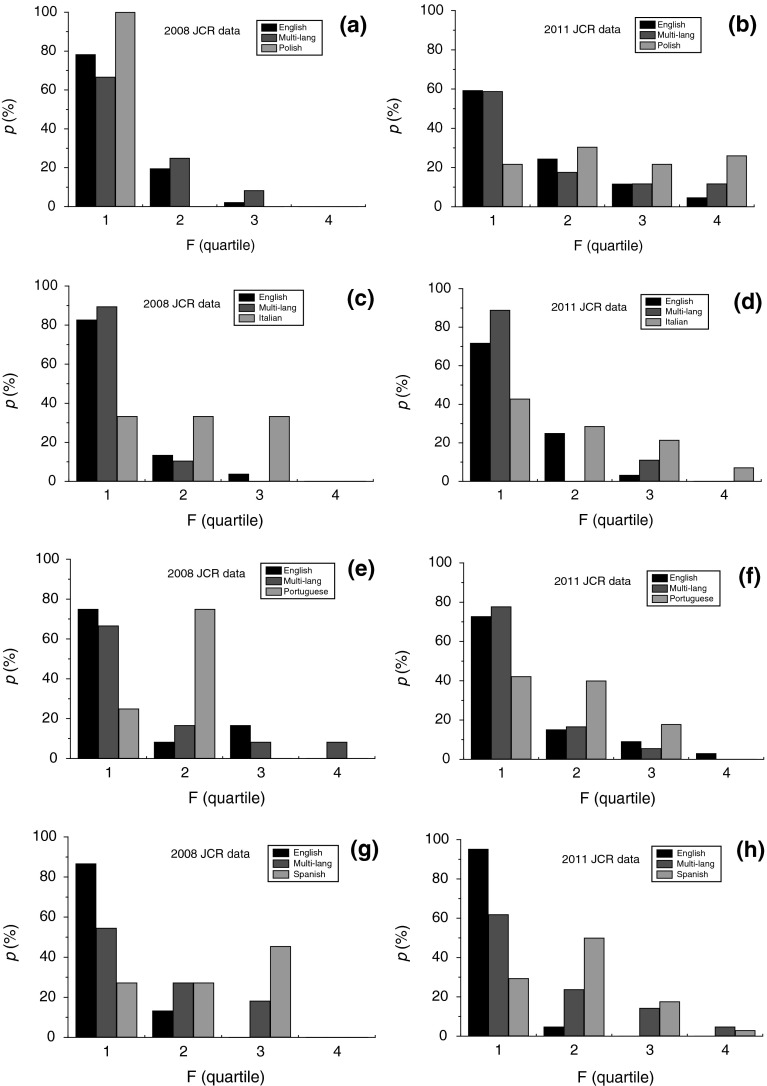
Histograms of relative participation *p* of four groups F of self-citation quartiles of journals published in **a**, **b** Poland, **c**, **d** Italy, **e**, **f** Brazil and **g**, **h** Spain. Data from **a**, **c**, **e**, **g** 2008 JCR, and **b**, **d**, **f**, **h** 2011 JCR

**Fig. 9:**
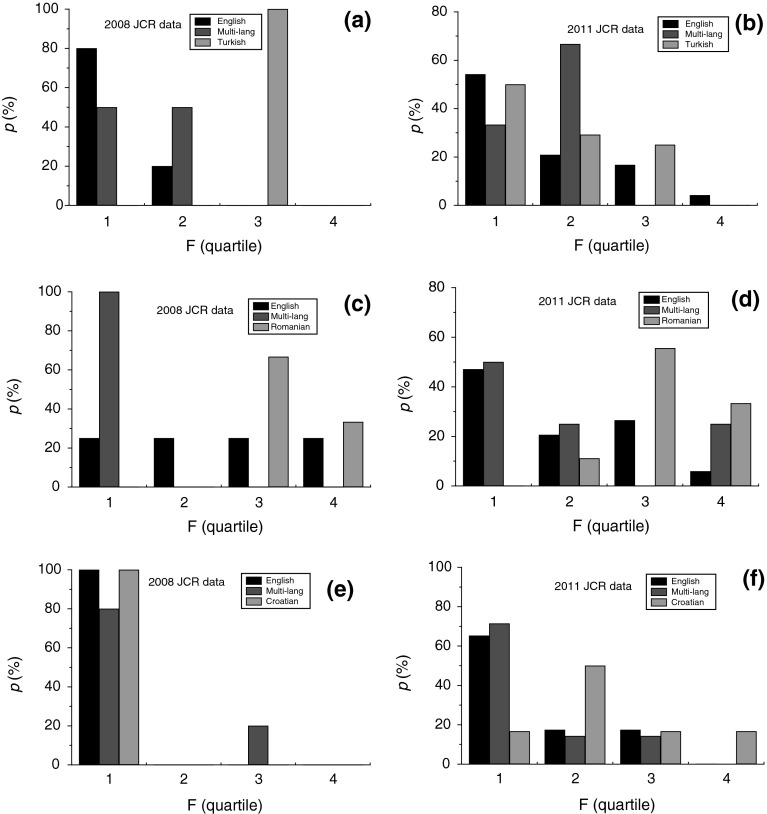
Histograms of relative participation *p* of four groups F of self-citation quartiles of journals published in **a**, **b** Turkey, **c**, **d** Romania and **e**, **f** Croatia. Data from **a**, **c**, **e** 2008 JCR, and **b**, **d**, **f** 2011 JCR

The effect of publication language of the journals published in different countries was analyzed from normalized fractions *p* of journals belonging to the four quartiles F1–F4. The normalized fraction *p* was calculated from the ratio of the number *N*_E_, *N*_N_ or *N*_ML_ of English-, national- or multi-language journals to their corresponding total number Σ*N*_E_, Σ*N*_N_ or Σ*N*_ML_ (for example: *p*_E_ = *N*_E_/Σ*N*_E_) in groups F1–F4 of self-citation quartiles. Histograms of the fractions *p* of journals belonging to the four quartiles of self-citations are presented in Figs. 8 and 9. Figures 8 and 9 show data for countries publishing relatively high and low number of journals classified according to the 2011 JCR database, respectively. We discuss below the general features of self-citations in these two classes of journals.

Figures 8 and 9 show that, with the exception of Romania, the fraction *p* of English-language journals published in different countries decreases with increasing self-citation quartile. The fractions of the journals in these countries are mainly limited to self-citation quartile F3 in the 2008 JCR database but they have gone down to F4 for most of the countries in the 2011 JCR database. The fraction *p* of multilingual journals published in Poland, Italy, Brazil and Spain also shows a decreasing trend with increasing self-citations. In the case of the remaining countries (Turkey, Czech Republic and Slovakia), it is difficult to establish any specific trends because of small number of multilingual journals published by them. In contrast to the trends of English-language and multilingual journals, the trends of changes in the fraction *p* of national-language journals published in the countries studied here with increasing self-citations are enormously different from each other, but a distinct difference in the self-citation behavior of journals indexed in the 2008 and 2011 JCR databases may be noted.

The national-language journals indexed in the 2008 JCR database from different countries is relatively small. Therefore, it is difficult to establish any self-citation trends for the national-language journals from the countries studied here. However, for the journals indexed in the 2011 JCR database one finds the following trends with increasing self-citation quartile: (1) the fraction *p* of non-English-language journals decreases for Italy (Fig. 8d), Brazil (Fig. 8f) and Turkey (Fig. 8b), (2) it remains distributed more or less uniformly over the first three or over all of self-citation quartiles for a country like Poland (Fig. 8b), and (3) it is distributed in such a way that its value is relatively low in self-citation quartile F1 and then changes nonuniformly in quartiles F2, F3 and F4 for countries like Spain (Fig. 8h) and Croatia (Fig. 9f). As judged from the self-citation quartiles of different countries, a clearly increased tendency of self-citations in national-language journals is seen in the histograms for practically all countries.

It should be noted that the self-citation behavior of English-, multi- and national-language journals published in Romania are completely different from their counterparts in other countries. For example, in contrast to the decreasing fraction *p* of English-language journals published in most countries with increasing self-citation quartile, the self-citation fraction *p* is equally distributed over all self-citation quartiles (Fig. 9c, d). In comparison with the trends of the distribution of *p* of the national-language journals published in other countries, the fraction *p* of Romanian-language journals mainly lies only in the highest self-citation quartiles F3 and F4.

From the above results it may be concluded that non-English language journals published in various countries usually have higher self-citations than English-language journals. A direct consequence of these high self-citations is to increase the values of their IF2. This tendency of high self-citations of journals, especially non-English-language journals, is associated with the national, regional or local nature of the subject matter of the papers published in them (Gonzalez-Alcaide et al. 2012), reluctance of authors of papers published in them to cite international literature due to language barrier and editorial policies of the journals (Bornmann et al. 2008; Gonzalez-Alcaide et al. 2012).

## High self-citation journals in different countries during 2008–2012

Journals with self-citations above 70 % published in the selected countries were compared to assess the trends of self-citations with increasing number of journals published in different countries. For this purpose, the relevant data collected from the 2008 and 2011 JCR databases are listed in Tables 7 and 8, respectively.

**Table 7:** Journals with high self-citations (SC) published in selected countries according to 2008 JCR

Country (*N*)	Journal title	Field	Lang.	IF2	SC (%)
Brazil (28)	Arq Bras Med Vet Zoo	Veterinary sci	ML	0.499	76
Turkey (8)	Klin Psikofarmakol B	Psychiatry	N	0.197	71
Romania (10)	Mater Plast	Mater sci (multidiscipl.)	N	0.873	77
	Carpath J Earth Env	Environ sci	E	0.286	100

**Table 8:** Journals with high self-citations (SC) published in selected countries according to 2011 JCR

Country (*N*)	Journal title	Field	Lang.	IF2	SC (%)
Poland (126)	Prz Elektroniczn	Engg, electr & elecron	N	0.244	77
	Arch Metall Mater	Metallurgy	N	0.487	84
	Sylwan	Forestry	N	0.159	78
	Och Sr	Environm engg	N	1.633	83
	Arch Acoust	Acoustics	E	0.847	73
	J Apic Sci	Entomology	E	0.674	79
	Postep Derm Alergol	Alergy, Dermatol.	N	0.357	73
	Arch Min Sci	Mining & miner proc	ML	0.350	91
	Acta Sci Pol-Hortoru	Horticulture	E	0.393	84
	Prz Menopauzalny	Obst & gynecology	N	0.190	85
	Videosurgery Miniinv	Surgery	E	1.000	86
	Rocz Ochr Sr	Environm engg	N	0.162	80
	Eksploat Niezawodn	Multidiscipl engg	E	0.333	70
	Kardiochir Torakochi	Surgery	N	0.135	76
	Drewno (wood/paper)	Mater sci	N	0.026	100
Italy (124)	Ofioliti	Geology	ML	1.125	72
	Nexus Netw J	History & philos of sci	E	0.070	75
	Veterinaria Cremona	Veterinary sci	N	0.062	100
	Acta Medica Mediterr	General medicine	N	0.031	100
Brazil (96)	Rev Bras Ensino Fis	Physical education	N	0.118	73
	Rev Bras Oftalmol	Ophthalmology	N	0.129	86
Spain (76)	Int Microbiol	Pharmacol/pharmacy	ML	1.407	85
	Rev Clin Esp	General/inter medicine	N	2.008	77
	Rev Int Androl	Andrology	N	0.213	100
	Aten Farm	Pharmacology	ML	0.082	85
Turkey (54)	Energy Educ Sci Tech	Environm engg, energy & fuels, chem engg	E	31.677	90
Romania (47)	Environ Eng Manag J	Environ engg	E	1.004	82
	Metal Int	Metallurgy	E	0.084	78
	Rev Rom Bioet	Medical ethics	N	0.683	80
	Rev Rom Mater	Construction engg, mater sci	ML	0.378	89
	Rev Roum Sc Tech-El	Electr engg	E	0.136	75
	Ind Textila	Mater sci (textiles)	N	0.291	76
	Gineco Ro	Gynecology	N	0.046	100
Croatia (36)	Teh Vjesn	Multidiscipl engg	N	0.347	71
	Promet –Zagreb	Transport	E	0.177	70
	Gradevinar	Civil engg	N	0.082	78

It may be noted from these tables that all countries have journals with very high self-citations, but the number of journals with high self-citations with reference to the total number of journals is relatively low in the 2008 JCR database in comparison with that in the 2011 JCR database. In general, the ratio of high self-citation journals to the total number of journals has increased significantly in the 2011 database from the 2008 database for all countries, but the value of the increase in the ratio of high self-citation journals for different countries varies enormously. Journals indexed in the 2008 JCR database from Romania, Turkey and Brazil show high self-citations but there are no high self-citation journals indexed in the 2008 JCR database from Poland, Italy, Spain and Croatia. However, in the 2011 JCR database, there are 12–15 % high self-citation journals from Poland and Romania, about 2 % from Brazil and Turkey, whereas it is intermediate for the remaining countries.

National-language journals have higher self-citations than English-language journals. However, it is a common observation that, irrespective of the publication language, journals devoted to very specialized scientific disciplines typically have relatively high self-citations. Among these disciplines are, for example, electrical and electronic engineering, metallurgy, environmental engineering, surgery, general and internal medicine, pharmacology and pharmacy, gynecology, entomology and multidisciplinary engineering.

The above observations suggest that, although all countries have highly self-cited journals, the proportion of the highly self-cited journals depends on the citation culture in different countries, the publication language of journals, their scientific discipline and the dissemination of their contents. The difference in the proportion of self-cited journals may also be attributed to the editorial policies of the journals published in these countries and the regional/local character of the contents of papers published in very specialized scientific disciplines of some of the journals.

## Conclusions

The following conclusions can be drawn from this study:

1. Analysis of data of two-year impact factor (IF2) against five-year impact factor (IF5) of different journals published in different countries according to the 2011 JCR database with the widest coverage of journals in the JCR databases revealed that IF2 ≈ IF5 for the journals published in Poland, Czech Republic and Croatia, IF2 > IF5 for the journals published from Turkey and Brazil, whereas IF2 < IF5 for the journals published in Romania. These relationships between IF2 and IF5 are related to the increasing, constant or decreasing number of citations received by the journals published in these countries. The unusual behavior of non-English-language journals published in Romania is mainly associated with the high self-citations of these journals which usually lie in categories Q3 and Q4.
2. English-language journals, as a rule, have higher impact factors than non-English-language journals.
3. All countries investigated in this study have journals with very high self-citations but the proportion of journals with high self-citations with reference to the total number of journals published in different countries varies enormously. National-language journals have higher self-citations than English-language journals.
4. Irrespective of the publication language, journals devoted to very specialized scientific disciplines have relatively high self-citations. Among these disciplines are, for example, electrical and electronic engineering, metallurgy, environmental engineering, surgery, general and internal medicine, pharmacology and pharmacy, gynecology, entomology and multidisciplinary engineering.
